# Extremely Low Frequency Electromagnetic Fields Facilitate Vesicle Endocytosis by Increasing Presynaptic Calcium Channel Expression at a Central Synapse

**DOI:** 10.1038/srep21774

**Published:** 2016-02-18

**Authors:** Zhi-cheng Sun, Jian-long Ge, Bin Guo, Jun Guo, Mei Hao, Yi-chen Wu, Yi-an Lin, Ting La, Pan-tong Yao, Yan-ai Mei, Yi Feng, Lei Xue

**Affiliations:** 1 State Key Laboratory of Medical Neurobiology, Department of Physiology and Biophysics, School of Life Sciences and Collaborative Innovation Centre for Brain Science, Fudan University, Shanghai, 200438, P.R.China; 2 Institute of Genetics, State Key Laboratory of Genetic Engineering, Fudan University, Shanghai, 200433, P.R.China; 3 Institutes of Brain Science, School of Life Sciences and State Key Laboratory of Medical Neurobiology, Fudan University, Shanghai, 200438, P.R.China; 4 Department of Critical Care Medicine, Shanghai General Hospital, Shanghai Jiaotong University, Shanghai, 200080, P.R.China

## Abstract

Accumulating evidence suggests significant biological effects caused by extremely low frequency electromagnetic fields (ELF-EMF). Although exo-endocytosis plays crucial physical and biological roles in neuronal communication, studies on how ELF-EMF regulates this process are scarce. By directly measuring calcium currents and membrane capacitance at a large mammalian central nervous synapse, the calyx of Held, we report for the first time that ELF-EMF critically affects synaptic transmission and plasticity. Exposure to ELF-EMF for 8 to 10 days dramatically increases the calcium influx upon stimulation and facilitates all forms of vesicle endocytosis, including slow and rapid endocytosis, endocytosis overshoot and bulk endocytosis, but does not affect the RRP size and exocytosis. Exposure to ELF-EMF also potentiates PTP, a form of short-term plasticity, increasing its peak amplitude without impacting its time course. We further investigated the underlying mechanisms and found that calcium channel expression, including the P/Q, N, and R subtypes, at the presynaptic nerve terminal was enhanced, accounting for the increased calcium influx upon stimulation. Thus, we conclude that exposure to ELF-EMF facilitates vesicle endocytosis and synaptic plasticity in a calcium-dependent manner by increasing calcium channel expression at the nerve terminal.

During the past few decades, considerable evidence has shown that non-thermal exposure to extremely low frequency electromagnetic fields (ELF-EMF)^1^ can induce biological changes both *in vivo* and *in vitro*, including gene expression^2^, tissue repair, and proliferation^3^, and it can also be conducive to the treatment of neurological disorders^4^,^5^. Despite accumulating experimental evidence suggesting significant biological effects, the underlying mechanisms are poorly understood. Laboratory studies have pointed to the initial effects of ELF-EMF being on the cellular level, especially the physiological properties of the cell membrane and channels^6^,^7^,^8^,^9^,^10^. Therefore, the neurons in the central nervous system are likely to be the most sensitive candidates, as exposure to ELF-EMF induces electrical fields and currents, which may excite or suppress neuronal activities through interactions with voltage-gated channels^11^,^12^.

Vesicle endocytosis, which couples to exocytosis and recycles exocytosed vesicles at the presynaptic nerve terminal, is a basic cellular mechanism that critically maintains synaptic transmission and plasticity^13^,^14^,^15^. Membrane fission caused by endocytosis also contributes to the homeostasis of the plasma membrane^14^,^16^. Although endocytosis fulfils such a crucial role in both physical and physiological aspects, very few studies regarding how ELF-EMF regulates this important cellular event in the central nervous system were reported and data from experimental studies are controversial^17^. In the present study, we directly measured vesicle exocytosis and endocytosis with accurate capacitance measurements^14^,^18^ at a central synapse, the calyx of Held. At calyces, several forms of endocytosis have been reported, including slow endocytosis^14^, rapid endocytosis^19^, endocytosis overshoot^20^, and bulk endocytosis^21^, and their underlying molecular mechanisms are different. Slow endocytosis is believed to be clathrin-dependent, whereas the others are not^22^. Whether exposure to ELF-EMF affects all forms of endocytosis is unknown.

Calcium, a mediator of intracellular signalling, has also been proposed to be affected by magnetic fields. In U937 cells, magnetic fields increase calcium influx and inhibit apoptosis^23^. In rat pituitary cells, exposure to 50 Hz magnetic fields increases the intracellular free calcium concentration^24^. However, this facilitation of intracellular calcium could not be independently replicated^25^,^26^,^27^. In addition, no effect on calcium influx was observed in isolated bovine chromaffin cells exposed to ELF-EMF up to 2.0 milliTesla (mT)^28^. In our study, we performed direct presynaptic measurements of calcium influx at calyces and suggested an increased calcium influx using different stimulation protocols. The post-tetanic potentiation (PTP), a calcium-dependent form of short-term plasticity, was also facilitated by the increased calcium influx after exposure to ELF-EMF. It is well accepted that calcium influx triggers exocytosis at nerve terminals, and we previously showed that calcium/calmodulin initiates all forms of endocytosis^14^,^18^,^19^, suggesting that the increased calcium influx accounts for the regulation of endocytosis after exposure to ELF-EMF. Thus, the voltage-gated calcium channels at the presynaptic nerve terminals may be the key factor underlying such ELF-EMF modulation^11^.

In this study, we provide for the first time direct evidence that ELF-EMF facilitates all forms of endocytosis and potentiates PTP^16^,^29^. Furthermore, the enhanced expression of calcium channels at the presynaptic nerve terminal, especially the P/Q type, increases calcium influx upon stimulation and facilitates vesicle endocytosis and synaptic plasticity. Our study provides novel insight into how ELF-EMF regulates neuronal activity and plasticity by increasing voltage-dependent calcium channels at the cellular level.

## Materials and Methods

### Electromagnetic field production

The system used to generate a magnetic field was similar as previously described^30^. Briefly, a 50 Hz magnetic field was generated by a pair of Helmholtz coils powered by a generator system producing the input pulse. The magnetic flux densities were adjusted to 1 mT and monitored by an electromagnetic field sensor with a digital multi-meter. The whole system could provide a uniform electromagnetic field for the animals within it.

### Slice preparation and electrophysiology

Postnatal day 8–10 (p8 – p10) old C57 mice of either sex were used in this study. The ELF-EMF exposure group was raised in the electromagnetic field from the day of birth (p0). Brain slice preparation was similar as previously reported^14^,^15^,^19^. Briefly, pups were decapitated and blocks of tissue containing the medial nucleus of the trapezoid body (MNTB) were placed in artificial cerebrospinal fluid (ACSF) solution (in mM: 125 NaCl, 25 NaHCO_3_, 3 myo-inositol, 2 Na-pyruvate, 2.5 KCl, 1.25 NaH_2_PO_4_, 0.4 ascorbic acid, 25 glucose, 3 MgCl_2_, and 0.05 CaCl_2_). Brain slices ~200 μm thick were prepared using a vibratome (VT 1200 s, Leica, Germany) and recovered in 37 °C with 95% O_2_ and 5% CO_2_ for 30 minutes before experiments. Electrophysiological recordings were made at room temperature (22–24 °C). Whole-cell capacitance measurements were made using the EPC-10 amplifier (HEKA, Lambrecht, Germany) with software lock-in amplifier. The presynaptic pipette (3.5–5 MΩ) solution contained (in mM): 125 Cs-gluconate, 20 CsCl, 4 Mg-ATP, 10 Na_2_-phosphocreatine, 0.3 GTP, 10 HEPES, and 0.05 BAPTA (pH 7.2, adjusted with CsOH). The series resistance (<10 MΩ) was compensated by 65% (lag 10 μs). For recordings of the PTP, a bipolar electrode was placed at the midline of the trapezoid body. A 0.1 ms, 2–20 V voltage pulse was applied to evoke an action potential, which induced an AMPA receptor-mediated excitatory postsynaptic current (EPSC) at the postsynaptic principal neuron. 1 mM kynurenic acid (KYN) was added to the bath solution to relieve AMPA receptor saturation and desensitization^19^,^31^. Voltage-clamp recordings of EPSCs and mEPSCs were made with an EPC 10 amplifier using pipettes (2–3 MΩ) containing (in mM): 125 K-gluconate, 20 KCl, 4 Mg-ATP, 10 Na_2_-phosphocreatine, 0.3 GTP, 10 HEPES, and 0.5 EGTA (pH 7.2, adjusted with KOH). The series resistance (<10 MΩ) was compensated by 90% (lag 10 μs). Statistical analysis used a t test unless otherwise noted, and means were presented as mean ± SE. All the methods were carried out in accordance with the approved guidelines and all animal experimental protocols were approved by the Animal Care and Use Committee of Fudan University.

### Western blot

Membrane proteins of calyces were extracted from both control and ELF-EMF exposure groups using Membrane and Cytosol Protein Extraction Kit (with membrane protein extraction reagent B, Beyotime Biotechnology, China) as described in previous study^32^. Protein samples were resolved on 10% SDS-PAGE and then transferred to PVDF membrane in transfer buffer (25 mM Tris, 192 mM glycine, and 20% methanol) for 20 min at 15 V. The membrane was blocked in TBS-T (20 mM Tris-HCl adjusted to pH 7.4, 500 mM NaCl, and 0.1% Tween 20) containing 5% non-fat milk at room temperature for 30 min and then probed with specific antibody overnight at 4 °C (pan and P/Q types: Abcam, USA; N type: Alomone, USA; R type: Sigma, USA). The membrane was blotted with horseradish peroxidase (HRP)-conjugated secondary antibody (Jackson, diluted 1:5000 in TBS-T with 5% non-fat milk) at room temperature for 30 min. After a final wash in TBS-T, the signal was detected using the ChemiDoc MP System (Bio-rad) according to the manufacturer’s instructions.

### Real-time PCR

Total RNA was extracted from calyces with TRIzol reagent (Invitrogen, USA). The concentration of RNA was measured using a NanoDrop 2000 spectrophotometer (Thermo Fisher, USA) with an OD_260_/OD_280_ ratio of 2.0. About 200 ng of total RNA was reverse-transcribed into cDNA using TransScript One-Step gDNA Removal and cDNA Synthesis SuperMix Kit (TransGen Biotech, China). All RNA and cDNA samples were stored at −70 °C before use.

Gene expression of all three calcium channel subtypes (P/Q, N, and R) was quantified by real-time PCR using SYBR^®^ Premix DimerEraser™ (Takara, Japan). The glyceraldehyde-3-phosphate dehydrogenase (GAPDH) gene was used as an internal control to normalise expression level of calcium channel genes. The primer sequences used are shown in Table 1.

## Results

### ELF-EMF increases mEPSC frequency but does not affect mEPSC amplitude

**Figure 1:**
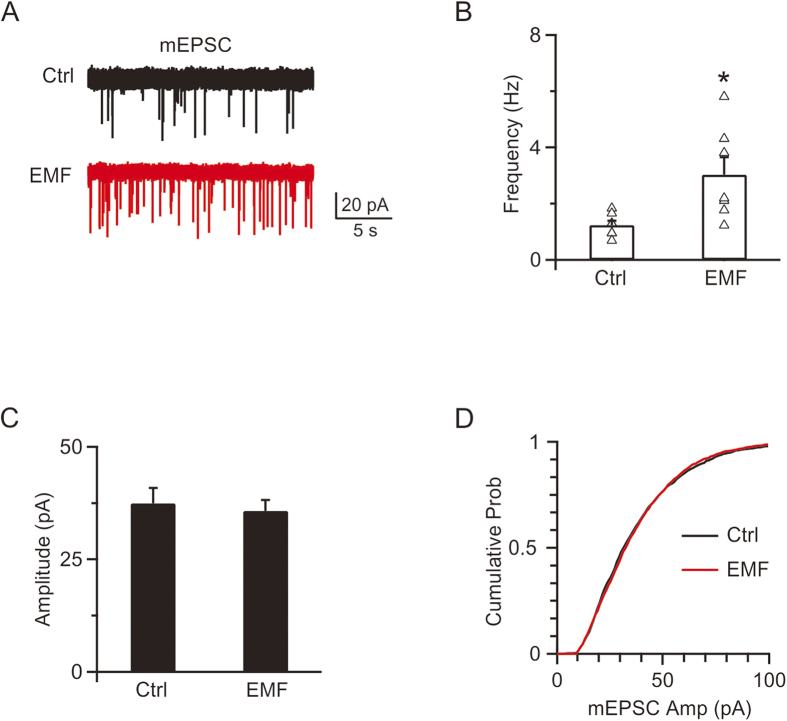
ELF-EMF exposure increases mEPSC frequency (**A**) Upper: sampled mEPSC in controls. Lower: sampled mEPSC in the ELF-EMF exposure group. (**B**) Mean of the mEPSC frequency distributions plotted for individual cells in the control and ELF-EMF exposure groups (triangle). (**C)** Mean of the mEPSC amplitude in the control (1363 events from 7 cells) and ELF-EMF exposure groups (1758 events from 7 cells). (**D**) The cumulative probability distribution in the control (black) and ELF-EMF exposure groups (red).

To evaluate how ELF-EMF regulates synaptic transmission, C57 mice were raised in a 50 Hz, 1 mT electromagnetic field from birth. Pups of p8–p10 from the control and ELF-EMF exposure groups were used to record mEPSCs. Whole cell voltage clamp recordings of mEPSCs are shown in Fig. 1A. In controls, the averaged mEPSC frequency and amplitude were 1.2 ± 0.2 Hz and 37.5 ± 3.4 pA, respectively (1363 events from 7 cells, Fig. 1B,C). However, exposure to ELF-EMF significantly increased the mEPSC frequency to 3.0 ± 0.6 Hz (n = 7, p < 0.05), though it did not affect the mEPSC amplitude (36.0 ± 2.4 pA, 1758 events from 7 cells; p = 0.7, Fig. 1B,C). The cumulative plots also confirmed that the amplitude distribution was not significantly different (p = 0.6, K-S test, Fig. 1D). The frequency increase in the ELF-EMF exposure group suggested a potential presynaptic mechanism^16^,^29^. However, the increase in presynaptic vesicle release frequency also raised the question of how neurons could maintain synaptic transmission with limitedly available vesicles at nerve terminals^15^,^22^,^33^.

### ELF-EMF facilitates both slow and rapid endocytosis

**Figure 2:**
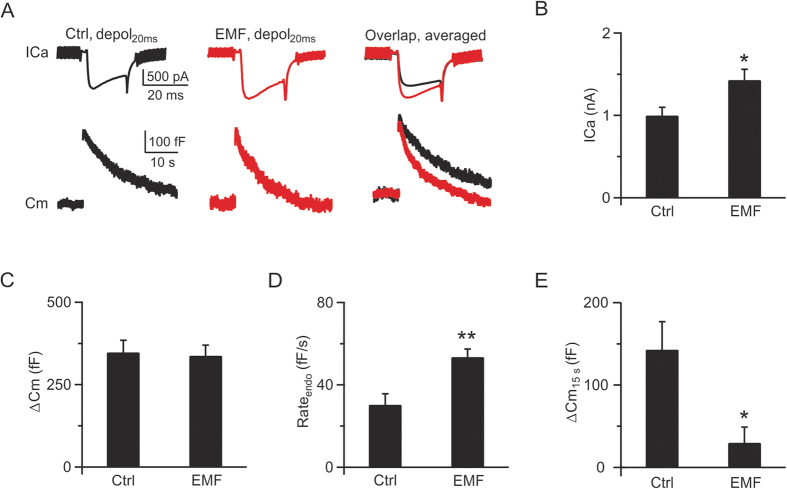
ELF-EMF exposure accelerates slow endocytosis (**A**) Left: Sampled presynaptic calcium current (ICa, upper) and membrane capacitance (Cm, lower) induced by a 20 ms depolarisation (depol_20ms_) in controls. Middle: Similar to Left, but for the ELF-EMF exposure group. Right: The averaged ICa (upper) and Cm (lower) from the control (n = 5, black) and ELF-EMF exposure groups (n = 5, red). (**B–E**) Statistics for ICa, ΔCm, Rate_endo_, and ΔCm_15s_ in the control and ELF-EMF exposure groups (*p < 0.05; **p < 0.01).

To further explore how ELF-EMF affects synaptic transmission, we examined synaptic vesicle exocytosis and endocytosis at presynaptic nerve terminals. We previously showed that different stimulation protocols induce different kinetics of exo-endocytosis^14^,^18^. A 20 ms depolarisation pulse (depol_20ms_, depolarised from −80 mV to +10 mV, same as below if not mentioned) can deplete the readily releasable pool (RRP) and induce a following clathrin-dependent, dynamin-dependent slow endocytosis^13^,^14^,^34^, whereas 10 depolarisation pulses of 20 ms at 10 Hz (depol_20msx10_) can induce a larger amount of exocytosis and an additional rapid form of endocytosis that depends on dynamin but not clathrin^18^,^19^,^34^,^35^. In p8 – p10 pups exposed to ELF-EMF, the depol_20ms_ induced a mean calcium influx of 1.4 ± 0.1 nA (n = 5; Fig. 2A, middle), which was larger than that in controls (1.0 ± 0.1 nA, n = 5, p < 0.05; Fig. 2A, left, 2B). The increased calcium influx did not significantly affect exocytosis (control: 348 ± 36 fF, n = 5; ELF-EMF: 338 ± 32 fF, n = 5; p = 0.8; Fig. 2A, right) because depol_20ms_ was strong enough to deplete the whole RRP, which was similar in both groups (Fig. 2C, see Supplementary Information II for detailed discussion on how EMF affects exocytosis and vesicle release probability). However, depol_20ms_ resulted in a significant difference in endocytosis after exposure to ELF-EMF. The mean endocytosis rate (Rate_endo_) was 54 ± 4 fF/s (n = 5) in the ELF-EMF exposure group, which was much faster than that in controls (30 ± 5 fF/s, n = 5, p < 0.01; Fig. 2D). The net capacitance increase 15 s after the stimulation (ΔCm_15s_) was 31 ± 20 fF (Fig. 2E) in the ELF-EMF exposure group, reflecting an almost full recovery. However, the ΔCm_15s_ in controls was larger (144 ± 28 fF, p < 0.05; Fig. 2E), further confirming the acceleration of slow endocytosis after ELF-EMF exposure.

**Figure 3:**
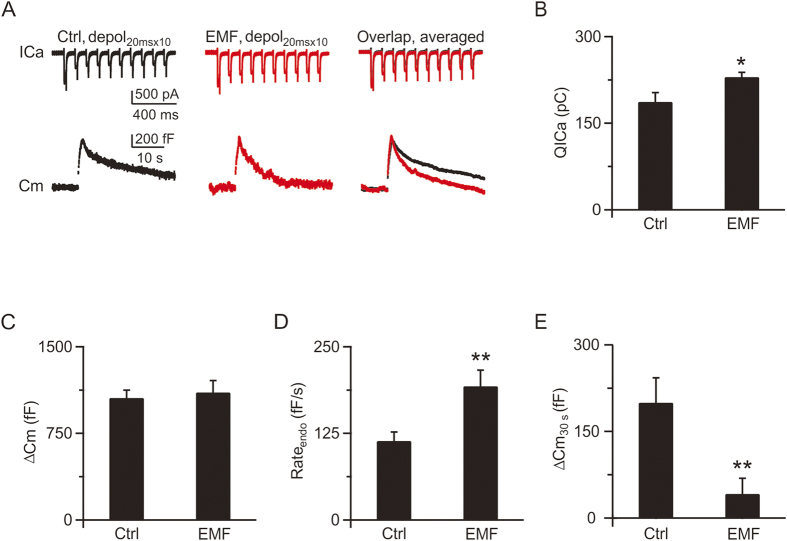
ELF-EMF exposure accelerates rapid endocytosis (**A**) Left: Sampled presynaptic calcium current (ICa, upper) and membrane capacitance (Cm, lower) induced by depol_20msx10_ in controls. Middle: Similar to Left, but for the ELF-EMF exposure group. Right: The averaged ICa (upper) and Cm (lower) from the control (n = 5, black) and ELF-EMF exposure groups (n = 6, red). (**B–E**) Statistics for QICa, ΔCm, Rate_endo_, and ΔCm_30s_ in the control and ELF-EMF exposure groups (*p < 0.05; **p < 0.01).

Next, we investigated whether ELF-EMF also affected the rapid form of endocytosis induced by intense stimulation^18^,^19^. In controls, depol_20msx10_ evoked a calcium influx (QICa) of 187 ± 16 pC (Fig. 3A, left, 3B) and a total capacitance jump (ΔCm) of 1057 ± 42 fF (n = 5, Fig. 3C), which was followed by a bi-exponential capacitance decay with time constants of 2.0 ± 0.1 s (amplitude, 250 ± 37 fF) and 18.0 ± 5.2 s, respectively (Fig. 3A, left and right). The initial endocytosis rate (Rate_endo_) measured within 2 s after depol_20msx10_ was 114 ± 13 fF/s, which reflects the speed of membrane retrieval caused by rapid endocytosis (Fig. 3D)^14^,^18^. In the ELF-EMF exposure group (Fig. 3A, middle), depol_20msx10_ evoked a larger calcium influx of 230 ± 8 pC (n = 6, p < 0.05; Fig. 3B) with a similar amount of exocytosis (1105 ± 103 fF, n = 6, p = 0.7; Fig. 3A, right, 3C). However, the endocytosis rate accelerated dramatically after depol_20msx10_. The capacitance decay could fit well with time constants of 1.1 ± 0.4 s (n = 6; amplitude, 364 ± 60 fF) and 14.0 ± 3.3 s, respectively (Fig. 3A, middle and right). The Rate_endo_ also increased to 193 ± 23 fF/s (n = 6, p < 0.01; Fig. 3D). Taken together, the increased Rate_endo_, decreased time constant (τ_rapid_), and increased size of the rapid component of endocytosis clearly indicated an acceleration of rapid endocytosis^18^. Furthermore, the increase in net capacitance 30 s after stimulation in the ELF-EMF exposure group was much smaller than in the control group (control: 200 ± 42 fF, ELF-EMF: 41 ± 27 fF, p < 0.01; Fig. 3E), which also confirmed the acceleration of endocytosis.

### ELF-EMF facilitates endocytosis overshoot and bulk endocytosis

**Figure 4:**
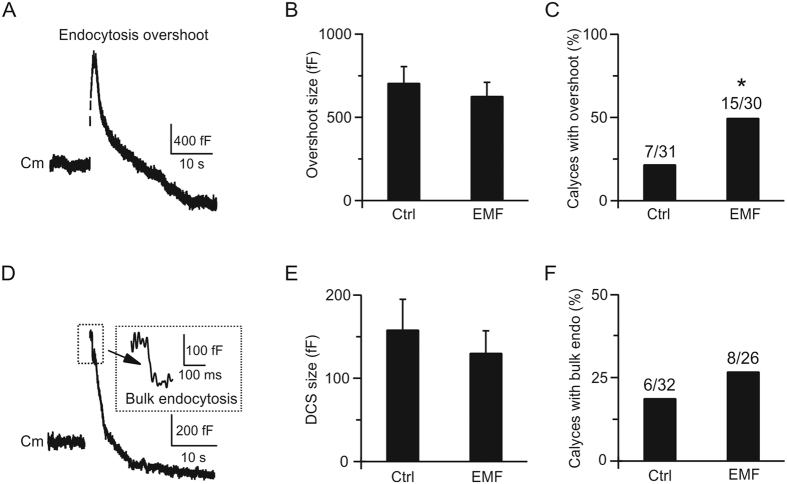
ELF-EMF exposure facilitates endocytosis overshoot and bulk endocytosis (**A**) Sampled Cm showing endocytosis overshoot induced by depol_50msx10_ with 5.5 mM extracellular calcium in controls. (**B,C**) Size and percentage of calyces showing endocytosis overshoot in the control (n = 31) and ELF-EMF groups (n = 30, *p < 0.05, chi-square test). (**D**) Sampled Cm showing bulk endocytosis induced by depol_50msx10_ with 5.5 mM extracellular calcium in controls. Inset, DCS in large scale. (**E,F**) Size and percentage of calyces showing bulk endocytosis overshoot in the control (n = 32) and ELF-EMF groups (n = 26).

Endocytosis overshoot, which retrieves more membrane than immediate exocytosis, has been reported in secretory cells and nerve terminals (Fig. 4A)^20^,^36^. We previously showed that the chance of observing endocytosis overshoot increases as calcium influx increases, from ~50% with 2 mM extracellular calcium to ~70% when calcium increases to 5.5 mM after 10 depolarisation pulses of 50 ms at 10 Hz (depol_50msx10_) in rats^20^. Having shown that exposure to ELF-EMF increases the calcium influx during depol_20ms_ or depol_20msx10_ stimulation, we investigated whether ELF-EMF also increases the fraction of calyces demonstrating endocytosis overshoot. In 7 out of 31 control calyces (23%), we observed significant endocytosis overshoot (>250 fF) after depol_50msx10_ (5.5 mM calcium in bath) with a mean size of 709 ± 96 fF (Fig. 4B,C). This proportion is much smaller than previously reported in rats (~70%)^20^ because the calcium influx during depol_50msx10_ was also much smaller than in rats. For example, the calcium influx induced by the first 50 ms depolarisation during depol_50msx10_ was only 92 ± 9 pC (n = 7) in mice but approximately 150 pC in rats^20^. Although the mean amplitude of endocytosis overshoot (709 ± 96 fF, n = 7) was smaller than in rats (~1000 fF), it was still roughly twice the RRP size in mice (348 fF from Fig. 2C), which was similar as reported in rats. In the ELF-EMF exposure group, the number of calyces demonstrating endocytosis overshoot dramatically increased (Fig. 4C, chi-square test, p < 0.05). In 15 out of 30 mice, we observed significant endocytosis overshoot with a mean size of 631 ± 80 fF (n = 15, p = 0.6; Fig. 4B,C). The increased proportion of calyces demonstrating endocytosis overshoot in the ELF-EMF exposure group was consistent with the larger calcium influx compared to controls (measured from the first 50 ms depolarisation during depol_50msx10_, control: 92 ± 9 pC, n = 7; ELF-EMF group: 119 ± 10 pC, n = 15; p < 0.01). However, the mean endocytosis overshoot size was not significantly different between the two groups, suggesting the same source from stranded vesicles at the presynaptic terminal in both groups^18^,^20^,^34^.

Bulk endocytosis, which directly retrieves a large piece of membrane from the presynaptic plasma membrane, has also been reported in neuronal cells (Fig. 4D)^18^,^21^,^37^. We previously reported that the frequency of bulk endocytosis increases with more intense stimulation, and buffering intracellular calcium with 70 mM EGTA abolishes bulk endocytosis^18^. Since ELF-EMF could increase the calcium influx, we further investigated whether it can also regulate bulk endocytosis. In 6 out 32 control calyces, we observed obvious bulk endocytosis after depol_50msx10_ (5.5 mM calcium in bath) with a mean size of 159 ± 36 fF (measured by the downward capacitance step, DCS; Fig. 4E,F). In 8 out of 26 calyces in the ELF-EMF exposure group, we observed obvious bulk endocytosis with a mean size of 131 ± 26 fF, which was similar to that of controls (p = 0.5, Fig. 4E,F). The proportion of calyces demonstrating bulk endocytosis after ELF-EMF exposure was only slightly higher than the proportion of controls (30% versus 18%) because the smaller calcium influx in mice during stimulation resulted in an even lower chance of detecting bulk endocytosis compared to rats. Nonetheless, the result was consistent with our hypothesis that a larger calcium influx increased the chance of detecting bulk endocytosis.

### ELF-EMF potentiates post-tetanic potentiation

**Figure 5:**
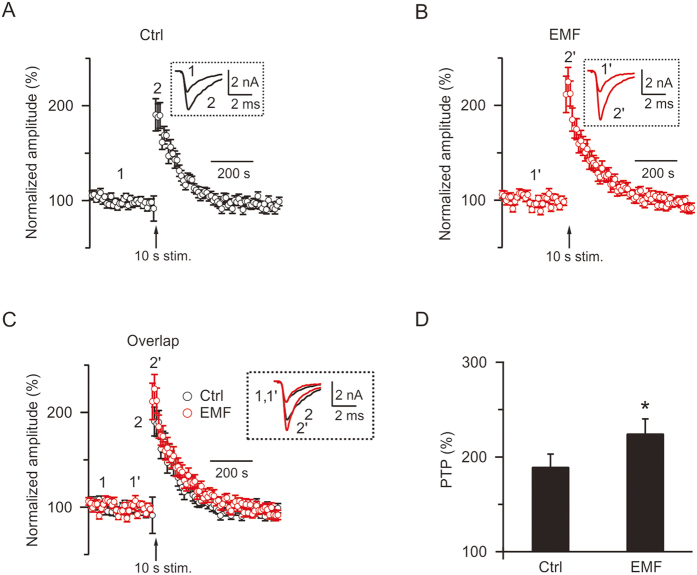
ELF-EMF potentiates post-tetanic potentiation (PTP) (**A**) Normalised EPSC amplitude change induced by a 100 Hz train for 10 s (Train_10s_) in the control group (n = 20). The arrow indicates the time the Train_10s_ was applied to induce PTP. Inset, sampled EPSC taken at times labelled. (**B**) Similar to A, but with the ELF-EMF exposure group (n = 23). (**C**) Similar to A and B, but with the control and ELF-EMF exposure groups in the same figure for comparison. (**D**) Amplitude of PTP (PTP%) in the control and ELF-EMF exposure groups (control: 190 ± 13%; ELF-EMF group, 225 ± 15%; *p < 0.05).

PTP, which is induced by a high-frequency train of action potential stimulation, has been reported at calyx of Held synapses as a form of short-term plasticity^16^,^29^,^31^,^38^. The amplitude of the PTP, which is represented by the normalised maximum EPSC after the stimulation train, is calcium-dependent^31^. Longer stimulation train increases PTP, whereas EGTA-AM suppresses PTP^16^,^31^. In the present study, we examined whether the increased influx of calcium caused by ELF-EMF exposure affects the PTP. First, we obtained a stable baseline of EPSCs by applying a brief stimulation (0.1 ms, 2–20 V) every 10 s for 300 s via a bipolar electrode positioned at the midline of the trapezoid body, and then applied a stimulation train at 100 Hz for 10 s (Train_10s_), inducing a robust PTP of the EPSC^16^,^29^. Shortly after the stimulation train, the EPSC amplitude was above baseline and peaked within 10–30 s (Fig. 5A,B). In controls, after the Train_10s_, the EPSC reached a maximum of 190 ± 13% of baseline (n = 20), which was similar to our previous report (Fig. 5A)^16^. However, in the ELF-EMF exposure group, the PTP amplitude was 225 ± 15% after Train_10s_ (n = 23, Fig. 5B,C), which is significantly higher than in controls (p < 0.05, Fig. 5D). This result is consistent with previous studies reporting that PTP is calcium-dependent and that longer stimulation train induced larger PTP^16^.

Although the increase in released vesicles dominate the peak of the PTP^31^,^39^, compound vesicle fusion has also been reported to contribute to the slow component of the PTP^16^,^29^. Here, two pieces of evidence ruled out this possibility after exposure to ELF-EMF: 1) we did not detect any mEPSC amplitude increase after ELF-EMF exposure (see Fig. 1), as compound fusion should increase mEPSC amplitude in parallel with the slow component of PTP^16^,^29^, and 2) the slow component of the PTP decay after Train_10s_ was not different between the two groups (control: τ = 112 ± 12 s, ELF-EMF group: τ = 114 ± 20 s, p = 0.8). Thus, we concluded that ELF-EMF potentiated the PTP by increasing the number of released vesicles but did not increase quantal size.

### ELF-EMF increases the calcium channels at the presynaptic nerve terminal

**Figure 6:**
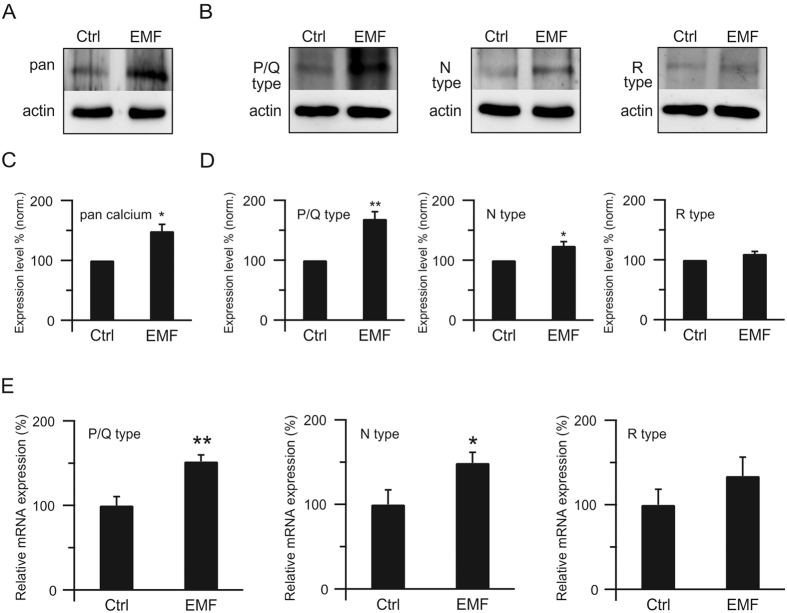
ELF-EMF increases calcium channels at the presynaptic nerve terminal (**A**) Western blot of pan calcium channel at the presynaptic terminal in the control (left) and ELF-EMF exposure groups (right). β-actin was used as a loading control. (**B**) Similar to A, but with P/Q (left), N (middle), and R (right) subtypes of calcium channels. (**C**) Expression level of pan calcium channel in the control (normalised) and ELF-EMF exposure groups (n = 4; **p < 0.01). (**D**) Similar to C, but with P/Q (left), N (middle), and R (right) subtypes of calcium channels (n = 4 for each; **p < 0.01; *p < 0.05). (**E**) Relative mRNA expression level of P/Q (left), N (middle), and R(right) subtypes of calcium channels (normalised to GAPDH, R subtype: p = 0.08; n = 9; **p < 0.01; *p < 0.05).

We have shown that ELF-EMF increases calcium influx upon stimulation, accelerating all forms of endocytosis and potentiating PTP. However, the underlying mechanisms that facilitate the calcium influx are unknown. Since calcium influxes through the calcium channels at the presynaptic nerve terminal during depolarisation^14^, we examined calcium channel expression at the presynaptic nerve terminal using calcium channel-specific antibodies. Western blot showed that pan calcium channel expression was much higher in the ELF-EMF exposure group than in controls (normalised, 149 ± 11%, n = 4, p < 0.05; Fig. 6A,C), which provided a structural guarantee for larger calcium influx during stimulation.

Three subtypes of calcium channels, P/Q, N, and R, are known to be expressed at the calyx of Held terminal^40^,^41^. Therefore, we further examined the expression levels of all three subtypes of calcium channels at the calyx of Held nerve terminal (Fig. 6B). Western blots showed that all three subtypes of calcium channels are expressed at higher levels in the ELF-EMF exposure group compared to controls, though the R-type did not reach significance (P/Q: 169 ± 12%, n = 4, p < 0.01; N: 124 ± 7%, n = 4, p < 0.05; R: 110 ± 4%, n = 4, p = 0.07; Fig. 6B,D), suggesting that all three subtypes could contribute to the acceleration of endocytosis and potentiation of PTP.

To investigate the mechanism underlying the increased expression of calcium channels at the presynaptic membrane after exposure to ELF-EMF, we used real-time PCR to examine the mRNA expression of the three subtypes of calcium channels (Fig. 6E). The relative mRNA expression levels of all three subtypes of calcium channels increased after exposure to ELF-EMF, though the R-type calcium channel did not reach significance (P/Q: p < 0.01; N: p < 0.05; R: p = 0.08; n = 9), suggesting that the increase in calcium channels at the presynaptic membrane was due to increased gene expression.

Previous studies have shown that the P/Q subtype calcium channel contributes 60–80% of the calcium current (N and R types contribute to less than 20% each)^42^ and dominates fast endocytosis at p8–p10 at calyx of Held synapses^43^. Therefore, we concluded that the increased calcium channel expression at the presynaptic membrane, especially the P/Q subtype calcium channel, accounts for the facilitation of endocytosis and potentiation of PTP. The up-regulated expression of N and R type channels may also contribute to the acceleration of the slow component of vesicle endocytosis and help potentiate PTP.

## Discussion

In this study, we report for the first time that exposure to ELF-EMF critically affects synaptic transmission and plasticity at calyx of Held synapses. With accurate presynaptic capacitance measurements, we provide direct evidence showing that exposure to ELF-EMF does not affect the RRP size during exocytosis, but dramatically accelerates all forms of vesicle endocytosis, including slow and rapid endocytosis, endocytosis overshoot, and bulk endocytosis (Figs 2, 3, 4). We also demonstrated that exposure to ELF-EMF potentiates synaptic transmission by increasing the amplitude of PTP, a form of short-term plasticity, but does not affect its time course^44^,^45^. We further investigated the underlying mechanisms by which exposure to ELF-EMF affects synaptic transmission and found that the enhanced expression of calcium channels at the presynaptic nerve terminal, mostly the P/Q subtype, accounts for the increased calcium influx upon stimulation, facilitating vesicle endocytosis and synaptic plasticity. These findings show crucial regulatory roles of ELF-EMF in synaptic transmission and plasticity in the central nervous system.

Endocytosis, an essential biological event that retrieves vesicular membrane and proteins, is important in preventing cells from swelling or shrinking and in maintaining synaptic transmission by preventing the depletion of synaptic vesicles^13^,^22^. Despite such important roles, studies regarding the effects of exposure to ELF-EMF on this cellular event are rare and controversial^17^. 50-Hz magnetic fields at 1 mT significantly stimulated the phagocytic activity of differentiated murine macrophages whereas 60-Hz magnetic fields resulted in no significant differences in the phagocytosis of *Candida albicans* by peritoneal murine macrophages^46^,^47^. Inhibitors of clathrin-dependent endocytosis were also reported to prevent the increase in endocytosis provoked by GSM-EMF (mobile phone EMF in particular) signals^17^. In the present study, we investigated the effects of ELF-EMF exposure on endocytosis in brain slices. All forms of endocytosis are accurately evaluated by direct capacitance measurements. Our findings suggest facilitation of all forms of endocytosis due to an increase in calcium influx.

Synaptic plasticity is important in neuronal circuit function^48^. PTP, a short-term plasticity of minutes induced by a high-frequency train of action potential stimulation, has been observed in calyces^16^,^29^,^39^. This form of short-term plasticity is reported to be calcium-dependent, which increases the number of vesicles released^38^,^39^,^49^. In the present study, we found that the increased influx of calcium also potentiates PTP (Fig. 5C). We previously showed that compound fusion between vesicles accounts for the mEPSC increase and slow component of PTP after the stimulation train^16^,^29^. As neither the increase in mEPSC amplitude nor changes in the slow component of PTP were observed after exposure to ELF-EMF, we concluded that compound fusion is not affected by ELF-EMF, which is consistent with the lack of changes in RRP size and exocytosis.

The biological effects of electromagnetic fields, especially the extremely low frequency fields, have been studied for more than fifty years and a huge amount of evidence has accumulated regarding the possible effects of ELF-EMF on living system^9^, including cancer^50^,^51^,^52^, immune cells^53^,^54^, bone cells^55^, and nerve cells^30^,^56^,^57^. However, there is still no general agreement on the relevant underlying mechanisms. Calcium, which acts as a messenger in many intracellular processes, such as differentiation, proliferation, and apoptosis, is strictly regulated in almost all cell types^58^, and many studies have shown that voltage-dependent calcium channels may account for the biological effects after exposure to EMF, such that calcium channel blockers could greatly lower the effects of ELF-EMF exposure^59^. It is well established that calcium triggers exocytosis and also we recently reported calcium initiates all forms of endocytosis^18^. Thus, our findings that the enhanced calcium channel expression, especially of the P/Q subtype, accelerates vesicle endocytosis and potentiates PTP may provide a new mechanism for how ELF-EMF regulates synaptic transmission at the cellular level in the central nervous system. The acceleration of endocytosis may facilitate synaptic strength, which may further regulate neuronal development, axonal branching, and refinement. The potentiation of PTP may also lead to strengthening the connection between neurons, which may further bolster the neuronal circuits^13^,^48^. Furthermore, enhanced calcium channel expression, especially of the P/Q subtype, after exposure to ELF-EMF may link many regulatory pathways that are calcium-dependent, such as the PKC pathway^29^,^39^ and calcium/calmodulin/calcineurin pathway^18^,^20^, which could induce more downstream regulatory factors. As efficient exo-endocytosis recycling is essential for brain function^13^, our findings may also offer new therapeutic insights for neurological disorders^60^.

How exposure to ELF-EMF increases more calcium channels at the presynaptic nerve terminal, and how these newly expressed channels are located in the right place at the active zone to trigger calcium influx upon stimulation are key questions that remain to be solved. Moreover, whether other proteins, such as SNARE proteins and synaptotagmin, are required during this process is still unknown. Understanding these questions would be of great interest in the future and help us resolve the details of the mechanisms underlying ELF-EMF-regulated neuronal communication.

## Additional Information

**How to cite this article**: Sun, Z.- *et al*. Extremely Low Frequency Electromagnetic Fields Facilitate Vesicle Endocytosis by Increasing Presynaptic Calcium Channel Expression at a Central Synapse. *Sci. Rep.* **6**, 21774; doi: 10.1038/srep21774 (2016).

## Supplementary Material

Supplementary Information

## Figures and Tables

**Table 1:** Primer sequences for real-time PCR.

Primer name	Sequence (5′ - 3′)
GAPDH-F	AGGTCGGTGTGAACGGATTTG
GAPDH-R	TGTAGACCATGTAGTTGAGGTCA
P/Q-F	CTTCAACTCCACCCTGATGGC
P/Q-R	AATGGCCATCATCTCCTTGCG
N-F	GTACCACCCCACAAACCTGAC
N-R	CAGAGGGTGGAACAGGGAAAC
R-F	ACTCTCATGTCACCACCGCTA
R-R	GTGTGGAGGTGAAGTGGACTG
