# Supplementary material for: Extremely Low Frequency Electromagnetic Fields Facilitate Vesicle Endocytosis by Increasing Presynaptic Calcium Channel Expression at a Central Synapse
Source: Sci Rep. 2016 Feb 18;6:21774. doi: 10.1038/srep21774 (PMC4757866; doi:10.1038/srep21774)
Supplement: Supplementary Information [file srep21774-s1.pdf]

Extremely Low Frequency Electromagnetic Fields Facilitate Vesicle Endocytosis by Increasing Presynaptic Calcium Channel Expression at a Central Synapse

Abbreviated title: Electromagnetic fields facilitate endocytosis by increasing calcium channel expression

Zhi-cheng Sun*[1], Jian-long Ge*[1], Bin Guo*[2], Jun Guo[1], Mei Hao[1], Yi-chen Wu[1], Yi-an Lin[1], Ting La[1], Pan-tong Yao[1], Yan-ai Mei[3], Yi Feng#[4] and Lei Xue#[1]

1. State Key Laboratory of Medical Neurobiology, Department of Physiology and Biophysics, School of Life Sciences and Collaborative Innovation Centre for Brain Science, Fudan University, Shanghai, P.R.China, 200438

2. Institute of Genetics, State Key Laboratory of Genetic Engineering, Fudan University, Shanghai, P.R.China, 200438

3. Institutes of Brain Science, School of Life Sciences and State Key Laboratory of Medical Neurobiology, Fudan University, Shanghai, P.R.China, 200438

4. Department of Critical Care Medicine, Shanghai General Hospital, Shanghai Jiaotong University, Shanghai, P.R.China, 200080

*: These authors contribute equally to this work.

#: corresponding author.

Supplementary Information

I. ELF-EMF exposure does not affect the basal EPSC amplitude

In Figure 5 of the main text, we showed that exposure to ELF-EMF could potentiate the PTP, a form of short-term plasticity. Because only normalized amplitude of EPSCs were shown, whether the basal EPSC amplitude was affected is unclear. We plotted the basal EPSC amplitude of both groups to address this issue (Fig. S1). With 1 mM kynurenic acid (KYN) in the bath solution, the EPSC amplitudes in the ELF-EMF exposure group showed no significant difference from the control group (control: 1.60 ± 0.20 nA, n = 20; ELF-EMF: 1.67 ± 0.21 nA, n = 23; p = 0.8), which suggests that the basal EPSC amplitudes are not affected by ELF-EMF exposure. Because KYN, an antagonist of the ionotropic AMPA receptors, was added to the bath solution to relieve AMPA receptor saturation and desensitization[1-3], which could reduce the EPSC amplitude in a dose-dependent manner, we only showed the normalized EPSC amplitudes in Figure 5 in the main text.

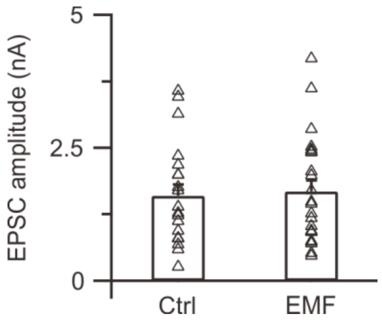

Figure S1. Exposure to ELF-EMF does not affect the basal EPSC amplitude

Basal EPSC amplitudes from each individual neuron were plotted from the control and ELF-EMF exposure groups. No significant difference was observed.

II. Exposure to ELF-EMF increases the probability of vesicle release

In the main text, we have shown that a 20 ms depolarisation pulse ($depol_{20ms}$) induced similar amounts of exocytosis in both control and ELF-EMF exposure groups

(Figure 2A, C). Previous studies have confirmed that $depol_{20ms}$ can deplete the readily releasable pool (RRP)[4,5]. However, whether ELF-EMF can affect the RRP size has not been rigidly tested. We performed new experiments to address this question. We applied stimulation pulses of various lengths (1, 2, 5, 10, 20, and 30 ms) from -80 to +10 mV to induce exocytosis in both groups[5]. In the control group, $depol_{20ms}$ induced a capacitance increase of 325 ± 42 fF (n = 7). Depolarisation pulses of 1, 2, 5 and 10 ms induced 4% ± 1% (n = 6), 13% ± 4% (n = 6), 42% ± 7% (n = 5), and 80% ± 5% (n = 7) of the capacitance jump induced by $depol_{20ms}$ measured at the same synapses, respectively (Fig. S2a, left). No further increase was observed when the step duration was increased to 30 ms (103% ± 6%, n = 6, p = 0.9), confirming that $depol_{20ms}$ could deplete the RRP.

In the ELF-EMF exposure group, $depol_{20ms}$ induced a capacitance increase of 324 ± 33 fF (n = 5), which was similar to that of the control group (p > 0.9). Depolarisation pulses of 1, 2, 5, and 10 ms induced 6% ± 1% (n = 6), 19% ± 1% (n = 6), 59% ± 3% (n = 6), and 92% ± 7% (n = 4) of the capacitance jump induced by $depol_{20ms}$ measured at the same synapses, respectively (Fig. S2a, righ). A 30 ms depolarisation could not induce a further capacitance increase (108% ± 3%, n = 5, p = 0.6), which confirmed that $depol_{20ms}$ could also deplete the RRP in the ELF-EMF group.

The results clearly showed that 1) $depol_{20ms}$ can sufficiently deplete the RRP in both the control and ELF-EMF exposure group, 2) the release probability increases with step duration, and 3) the release probability is higher in the ELF-EMF exposure group than controls with the same stimulation step due to the increased calcium influx after ELF-EMF exposure (Fig. S2b).

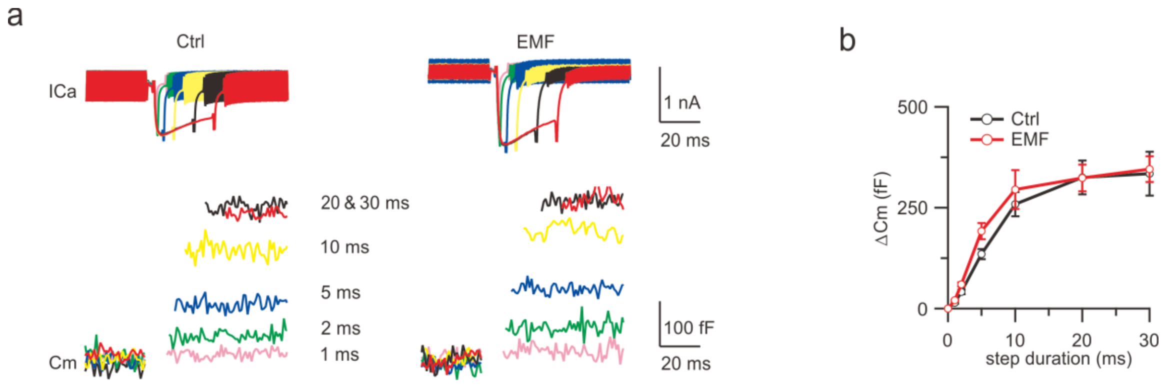

Figure S2. Exposure to ELF-EMF increases the release probability

a) Left, sampled ICa and Cm induced by depolarisation steps from –80 to +10 mV of various lengths (1, 2, 5, 10, 20, and 30 ms) in the control group. The time scale applied to all traces. Right, similar to Left, but with the ELF-EMF exposure group.

b) Relationship between ΔCm and the step duration of depolarisation (control, black; EMF, red).

III. Exposure to ELF-EMF does not affect the action potential

Whether other ion channels, such as sodium and potassium channels, are also affected after exposure to ELF-EMF is unclear. We address this question by recording the action potential wave form at the presynaptic nerve terminal of calyces[6]. A 3 ms step current of 400 pA was applied under the current clamp mode to induce the action potential[7]. The action potential wave did not exhibit a significant difference in either amplitude (control: 102 ± 3 mV, n = 4; ELF-EMF group: 104 ± 3 mV, n = 4; p = 0.6) or half-width (control: 0.48 ± 0.04 ms, n = 4; ELF-EMF group: 0.44 ± 0.02 ms, n = 4; p = 0.5) in the control and ELF-EMF exposure groups, which suggests that the voltage-gated sodium and potassium channels are largely unaffected in generating action potential (Fig. S3).

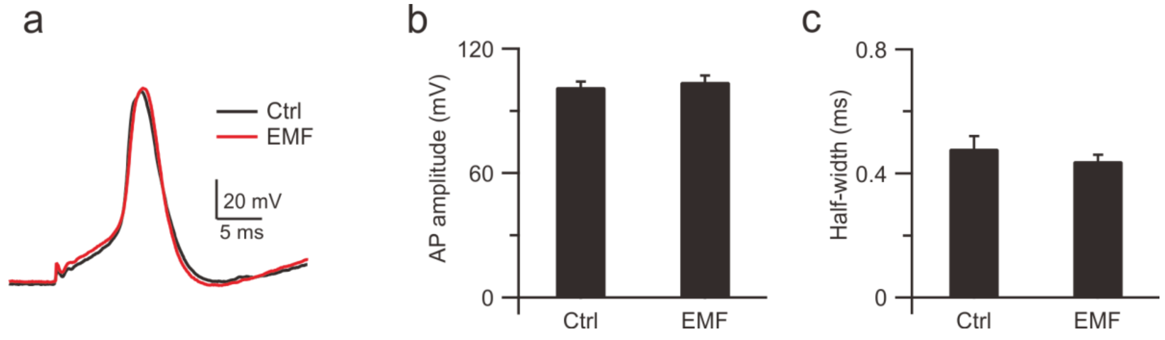

Figure S3. Exposure to ELF-EMF does not affect the action potential amplitude and half-width

a) Sampled action potential wave form recorded in the current clamp mode from the control (black) and ELF-EMF exposure groups (red).

b) Action potential amplitude in the control and ELF-EMF exposure groups.

c) The half-width of the action potential in the control and ELF-EMF exposure groups.

IV. Acute increase of calcium influx accelerates endocytosis

In the main text, we concluded that exposure to ELF-EMF up-regulates calcium channel expression at the presynaptic membrane and facilitates vesicle endocytosis. To further strengthen the conclusion, we acutely increased the calcium influx in controls to see whether it could have similar effect to the ELF-EMF exposure group.

We increased the calcium influx two ways: 1) we increased the extracellular calcium concentration from 2 mM to 4 mM (Fig. S4), and 2) we increased the stimulation duration from 20 ms to 50 ms in the same cells (Fig. S5). First, with $depol_{20ms}$, increasing of the extracellular calcium concentration to 4 mM resulted in increased calcium influx from 20 $\pm$ 1 pC to 31 $\pm$ 3 pC (n = 4, $p < 0.05$, Fig. S4a, b), and increased endocytosis rate from 30 $\pm$ 3 fF/s to 51 $\pm$ 6 fF/s (n = 4, $p < 0.05$, Fig. S4d). Increasing the stimulation duration from $depol_{20ms}$ to $depol_{50ms}$ also significantly increased the calcium influx to 39 $\pm$ 3 pC (n = 3, $p < 0.05$, Fig. S5a, b) and accelerated the endocytosis rate (65 $\pm$ 3 fF/s, p <

0.01, Fig. S5d). Second, with $depol_{20msx10}$, increasing of the extracellular calcium concentration to 4 mM resulted in increased calcium influx from 162 ± 15 pC to 256 ± 16 pC (n = 4, p < 0.01, Fig. S4e, f), and increased endocytosis rate from 102 ± 10 fF/s to 212 ± 39 fF/s (n = 4, p < 0.05, Fig. S4h). Increasing the stimulation duration from $depol_{20msx10}$ to $depol_{50msx10}$ also significantly increased the calcium influx to 294 ± 4 pC (n = 3, p < 0.01, Fig. S5e, f) and accelerated the endocytosis rate (193 ± 16 fF/s, p < 0.01, S5h). Exocytosis was not significantly different in these scenarios (Figs. S4c, g; S5c, g). Taken together, our new experiments confirmed that vesicle endocytosis was accelerated after an acute increase in calcium influx, which is consistent with the results in the ELF-EMF exposure group.

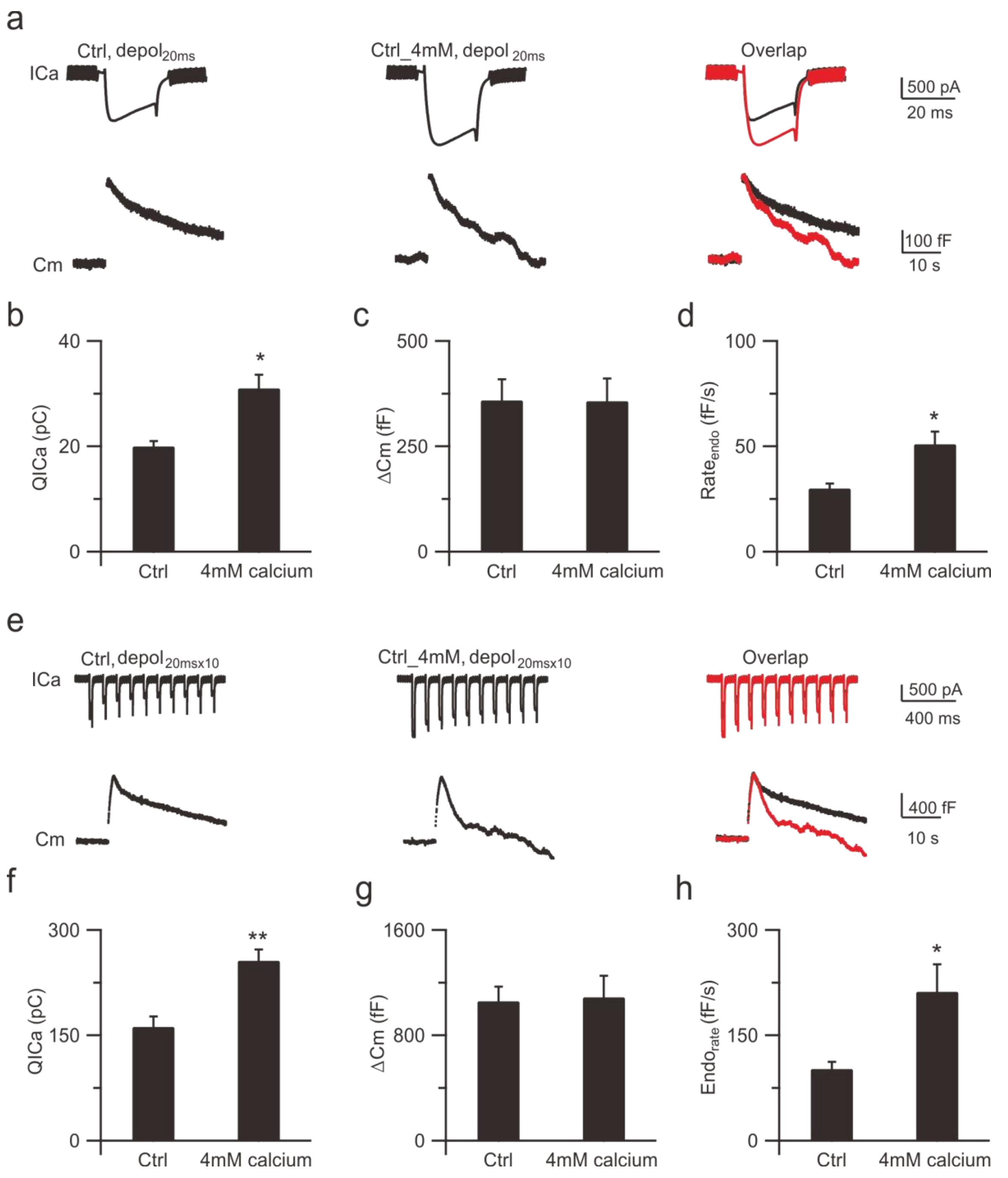

Figure S4. Acute increase of calcium influx with 4 mM extracellular calcium accelerates endocytosis

a) Left: Sampled presynaptic calcium current (ICa, upper) and membrane capacitance (Cm, lower) induced by $depol_{20ms}$ with 2 mM extracellular calcium (ctrl). Middle: Similar to Left, but with 4 mM calcium in the bath solution in the same cell. Right: Overlap of Left and Middle.

b-d) Statistics for QICa, ΔCm, and $Rate_{endo}$ with 2 mM (ctrl) and 4 mM extracellular calcium (*: $p < 0.05$).

e) Similar to a, but with stimulation of $depol_{20msx10}$.

f-h) Similar to b-d, but with stimulation of $depol_{20msx10}$ (*: $p < 0.05$; **: $p < 0.01$).

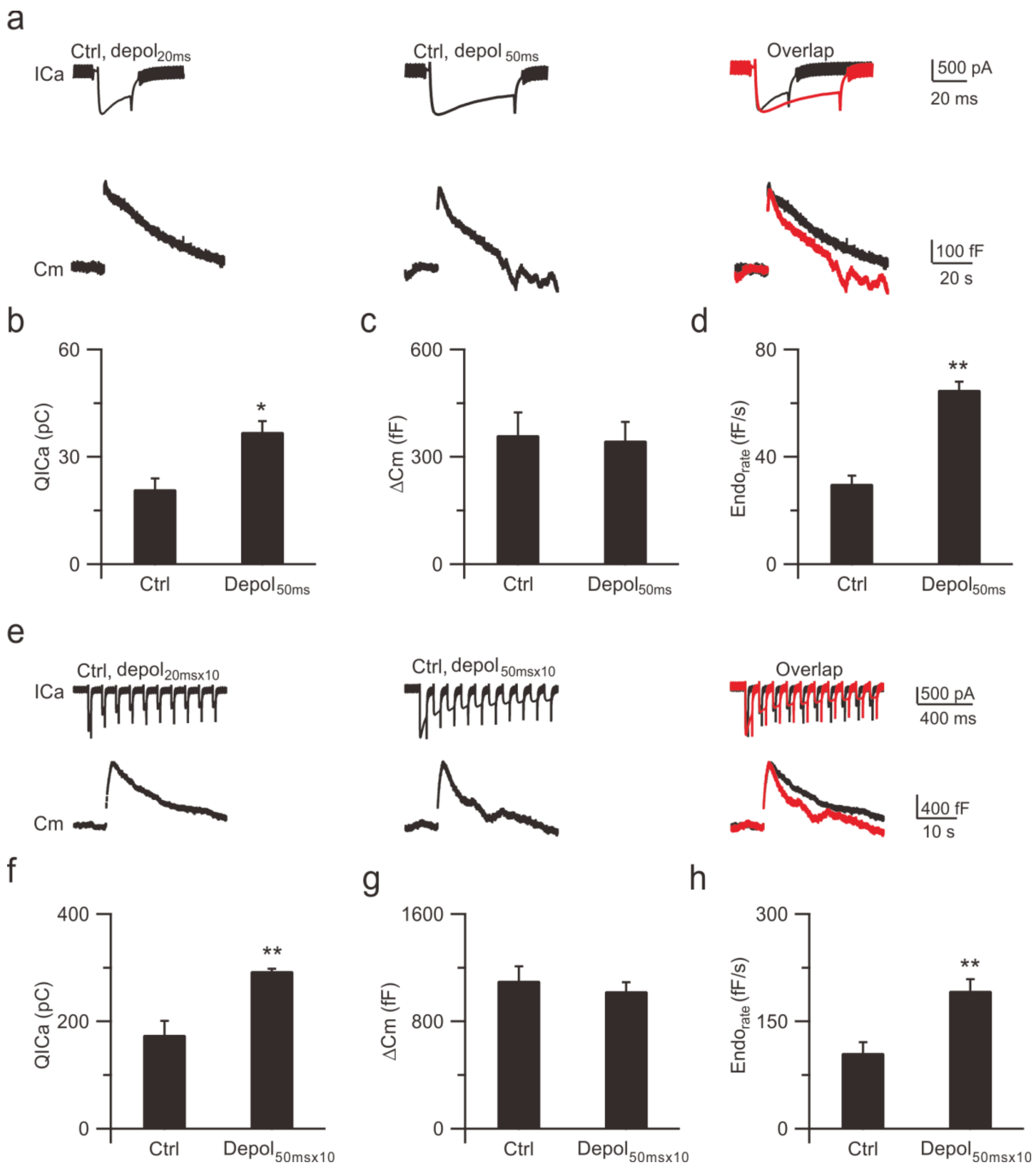

Figure S5. Acute increases of calcium influx with $depol_{50ms}$ and $depol_{50mx10}$ accelerate endocytosis

a) Left: Sampled presynaptic calcium current (ICa, upper) and membrane capacitance (Cm, lower) induced by $depol_{20ms}$ with 2 mM extracellular calcium (ctrl). Middle: Similar to Left, but with $depol_{50ms}$ in the same cell. Right: Overlap of Left and Middle.

b-d) Statistics for QICa, ΔCm, and $Rate_{endo}$ with $depol_{20ms}$ and $depol_{50ms}$ (*: $p < 0.05$; **: $p < 0.01$).

e) Similar to a, but with stimulation of $depol_{50msx10}$.

f-h) Similar to b-d, but with stimulation of $depol_{50msx10}$ (**: $p < 0.01$).

V. Original Western blot results

We showed calcium channel expression levels in main text (Fig. 6A, B) and found that the enhanced calcium channel expression at the presynaptic nerve terminal, especially the P/Q subtype, accounts for the influx calcium upon stimulation, acceleration of all forms of endocytosis, and potentiation of PTP. Here we provided the original Western blot results (Fig. S6).

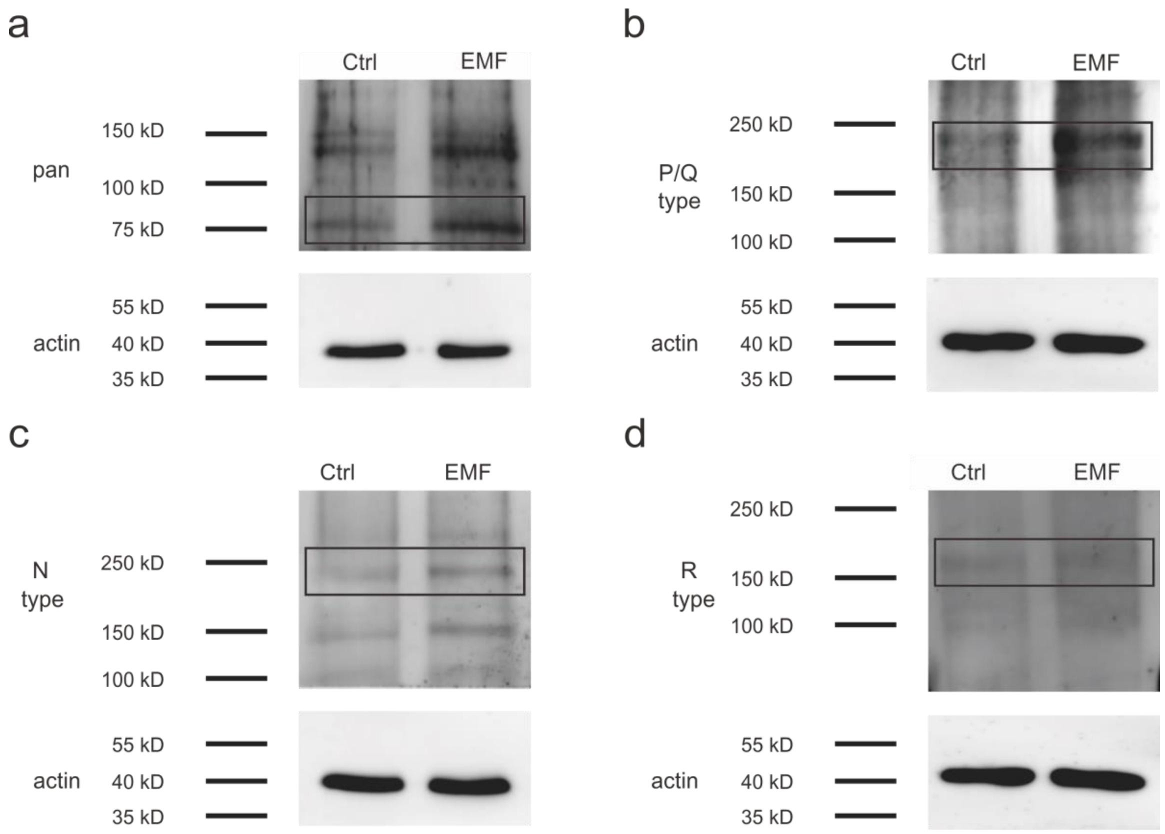

Figure S6. ELF-EMF increases calcium channel expressions at the presynaptic nerve terminal

a) Original sampled Western blot of pan calcium channel at the presynaptic terminal in the control (left) and ELF-EMF exposure groups (right). β-actin was used as a loading control.

b-d) Original sampled Western blots of P/Q, N, and R subtype calcium channels at the presynaptic terminal in the control (left) and ELF-EMF exposure groups (right). The black square indicated shows the band used in main text. β-actin was used as a loading control.

Reference List

1 Wu, W., Xu, J., Wu, X. S. & Wu, L. G. Activity-dependent acceleration of endocytosis at a central synapse. *J Neurosci* **25**, 11676-11683 (2005).

2 Korogod, N., Lou, X. & Schneggenburger, R. Presynaptic Ca2+ requirements and developmental regulation of posttetanic potentiation at the calyx of Held. *J Neurosci* **25**, 5127-5137 (2005).

3 Wong, A. Y., Graham, B. P., Billups, B. & Forsythe, I. D. Distinguishing between presynaptic and postsynaptic mechanisms of short-term depression during action potential trains. *J.Neurosci.* **23**, 4868-4877 (2003).

4 Xue, L. *et al.* A membrane pool retrieved via endocytosis overshoot at nerve terminals: a study of its retrieval mechanism and role. *J Neurosci* **32**, 3398-3404, doi:10.1523/JNEUROSCI.5943-11.2012 (2012).

5 Xue, L. *et al.* Voltage-dependent calcium channels at the plasma membrane, but not vesicular channels, couple exocytosis to endocytosis. *Cell reports* **1**, 632-638, doi:10.1016/j.celrep.2012.04.011 (2012).

6 Kushmerick, C. *et al.* Retroinhibition of presynaptic Ca2+ currents by endocannabinoids released via postsynaptic mGluR activation at a calyx synapse. *J Neurosci* **24**, 5955-5965, doi:10.1523/JNEUROSCI.0768-04.2004 (2004).

7 Wu, X. S., Sun, J. Y., Evers, A. S., Crowder, M. & Wu, L. G. Isoflurane inhibits transmitter release and the presynaptic action potential. *Anesthesiology* **100**, 663-670 (2004).
